# Why Should ACT Work When CBT Has Failed? a Study Assessing Acceptability and Feasibility of Acceptance and Commitment Therapy (ACT) for Paediatric Patients With Chronic Fatigue Syndrome/myalgic Encephalomyelitis (CFS/ME)

**DOI:** 10.1192/bjo.2022.209

**Published:** 2022-06-20

**Authors:** Jamie Leveret, Jen Starbuck, Kate Chapple, Sam Gubb, Hannah Kwuo, Sarah Burge, Morain Li, Philippa Clery, Esther Crawley

**Affiliations:** 1 University of Bristol, Bristol, United Kingdom; 2 Royal United Hospital Bath, Bath, United Kingdom

## Abstract

**Aims:**

Paediatric chronic fatigue syndrome/myalgic encephalomyelitis (CFS/ME) effects 0.5–3.28% of children. NICE guidance recommends Activity Management, Graded Exercise Therapy or Cognitive Behavioural Therapy for fatigue (CBT-f). Approximately 15% of patients do not achieve full recovery within one year with current treatments. Acceptance and Commitment Therapy (ACT) is an effective treatment in many chronic illnesses. There are no studies investigating ACT for paediatric CFS/ME. This feasability study aimed to assess if ACT is a feasible and acceptable alternative treatment when current treatment has not led to recovery.

**Methods:**

This feasability cohort study aimed to enrol a minimum of 12 participants aged 11–18 yearswith CFS/ME attending the Royal United Hospitals Bath NHS Foundation Trust Specialist Paediatric CFS/ME Service, who were still symptomatic after 12 months or 12 sessions of standard treatment and were offered six to 12 sessions of ACT. Retention and recruitment data were analysed. Participants were asked to complete questionnaires before, during and after treatment. A selection of participants and their parents were interviewed about their experience of the study. Interviews were analysed using thematic analysis.

**Results:**

19 participants (95% of those approached) were recruited. Only 4 participants of this hard-to-reach group did not complete treatment.

In almost all sessions participants reported that they felt ‘totally’ listened to in post session questionnaires (31/33 sessions).

Preliminary interviews (n = 12) indicate acceptability of ACT, with all young people and their parents stating that they thought ACT should be offered to this population. Participants particularly commented that the absence of thought challenging (used in CBT-f) was a positive element of ACT. Participant's openness to try new approaches and altruistic desire to be in a study was noted.

**Conclusion:**

Recruitment data indicate that it is feasible to recruit and retain 11–18-year-olds with CFS/ME to a study offering ACT. Interviews with participants and parents were broadly positive suggesting ACT is an acceptable treatment in this population.

Results indicated that it is both feasible and acceptable to offer ACT to 11–18-year-olds with CFS/ME using this protocol, supporting the prospect of an RCT in this area.

